# Channel Estimation in UAV-Assisted OFDM Systems by Leveraging LoS and Echo Sensing with Carrier Aggregation

**DOI:** 10.3390/s25206392

**Published:** 2025-10-16

**Authors:** Zhuolei Chen, Wenbin Wu, Renshu Wang, Manshu Liang, Weihao Zhang, Shuning Yao, Wenquan Hu, Chaojin Qing

**Affiliations:** 1 Electric Power Research Institute of State Grid Fujian Electric Power Co., Ltd., Fuzhou 350007, China; wangrenshu_1@126.com (R.W.); amanda498124338@gmail.com (M.L.); tedtom@126.com (W.Z.); yao_shuning@163.com (S.Y.); 2 School of Electrical Engineering and Electronic Information, Xihua University, Chengdu 610039, China; huwenquan@stu.xhu.edu.cn (W.H.); qingchj@mail.xhu.edu.cn (C.Q.)

**Keywords:** unmanned aerial vehicle (UAV), channel estimation (CE), echo sensing, carrier aggregation (CA), delay-Doppler (DD)

## Abstract

Unmanned aerial vehicle (UAV)-assisted wireless communication systems often employ the carrier aggregation (CA) technique to alleviate the issue of insufficient bandwidth. However, in high-mobility UAV communication scenarios, the dynamic channel characteristics pose significant challenges to channel estimation (CE). Given these challenges, this paper proposes a line-of-sight (LoS) and echo sensing-based CE scheme for CA-enabled UAV-assisted communication systems. Firstly, LoS sensing and echo sensing are employed to obtain sensing-assisted prior information, which refines the CE for the primary component carrier (PCC). Subsequently, the path-sharing property between the PCC and secondary component carriers (SCCs) is exploited to reconstruct SCC channels in the delay-Doppler (DD) domain through a three-stage process. The simulation results demonstrate that the proposed method effectively enhances the CE accuracy for both the PCC and SCCs. Furthermore, the proposed scheme exhibits robustness against parameter variations.

## 1. Introduction

### 1.1. Background

Unmanned aerial vehicles (UAVs) have been widely deployed in numerous civil applications, such as power inspection, disaster rescue, agricultural monitoring, and logistics delivery, as well as specialized scenarios, including military reconnaissance, owing to their advantages in flexible deployment, high mobility, and extensive coverage [1,2]. With the evolution from 5th-Generation (5G) to 6th-Generation (6G) networks, UAV-assisted communication systems, which enable rapid deployment of temporary communication infrastructures and enhanced coverage quality, have become a critical component of the space–air–ground–sea integrated network [3]. In UAV-assisted communication systems, the performance of communication links is directly determined by the accuracy of channel estimation (CE), thereby affecting flight safety and mission completion [4,5,6,7]. On the one hand, the high mobility of UAVs results in rapid time-varying channels. On the other hand, applications such as high-definition video transmission and multi-UAV coordination impose more stringent requirements for system bandwidth [8]. Notably, UAV-assisted communication systems exhibit dominant line-of-sight (LoS) links and echo-based sensing information. The exploitation of these characteristics and sensing information offers significant potential to mitigate the impact of rapid channel variations caused by high UAV mobility. To address the issue of bandwidth demand, carrier aggregation (CA) technology efficiently integrates discrete spectrum resources [9,10,11,12]. It not only enhances the capacity of UAV-assisted communication systems but also provides opportunities for developing LoS and echo sensing information-based CE methods.

### 1.2. Related Works

In UAV-assisted communication systems, orthogonal frequency division multiplexing (OFDM) modulation has attracted significant attention due to its robust ability to mitigate multipath interference [5,6,7,13]. For UAV-assisted OFDM systems, considerable research has been conducted on CE for ground base stations (gBSs) [5,6,7,14,15,16,17]. In [5], tailored tensor models are employed across different UAV-assisted communication scenarios to achieve joint CE and symbol detection (SD). To enhance bandwidth efficiency in OFDM-based UAV communication systems operating in time-varying environments, a deep learning-based pilot-free approach for joint channel and carrier frequency offset equalization is proposed in [6]. Meanwhile, a multi-resolution deep neural network-based CE method is proposed in [7] for UAV-assisted OFDM systems to enhance both computational efficiency and prediction performance. To address the beam squint issue in high-frequency bands, a gridless compressed sensing (CS)-based CE method is proposed for UAV-assisted communications in [14]. For massive multiple-input multiple-output (MIMO) UAV-assisted communication systems with a single radio-frequency-chain architecture, a hybrid parametric/non-parametric CE scheme incorporating UAV state-space information is proposed in [15]. While the aforementioned methods offer valuable insights into CE for UAV-assisted OFDM systems, they generally overlook the dominant LoS characteristics inherent in UAV scenarios. Inspired by integrated sensing and communication (ISAC), an LoS sensing-enhanced CE method is proposed in [16] for UAV-assisted OFDM systems to improve estimation accuracy by applying a denoising threshold for LoS/non-line-of-sight (NLoS) detection. For UAV emergency communications, a pilot-based ISAC system is developed in [17], which enhances communication reliability while simultaneously achieving superior ranging accuracy compared to conventional schemes. Although prior studies have explored ISAC-enabled UAV communication designs [18,19,20], the research on sensing-assisted CE schemes remains limited, impeding further enhancement of CE accuracy in UAV-assisted OFDM systems.

In ISAC systems, several studies have focused on leveraging sensing-based parameters to enhance CE performance [21,22,23,24,25,26,27,28,29,30]. In [21], roadside units employ ISAC signals to serve downlink information transmission and channel parameter prediction. This approach designs a downlink beamforming method to mitigate channel interference, thereby enabling vehicles to directly detect information without performing CE. To address the lack of signal processing capability at an intelligent reflecting surface (IRS) as well as the mutual interference between sensing and communication signals, a deep learning-based approach is explored in [22] for CE in IRS-assisted ISAC systems. By leveraging joint burst sparsity and pilot beamforming gain, a two-stage joint scheme for beam steering optimization, target detection, and CE is proposed in [23], enhancing both detection and estimation performance. To reduce communication overhead and enhance efficiency, the application potential of extreme learning machines in IRS-assisted multiuser ISAC systems is demonstrated in [24]. In [25], a closed-form solution for minimum mean square error (MMSE) CE is derived under the constraints of communication CE and a radar ambiguity function by leveraging reference signals for radar sensing. Leveraging the correlation between channel and sensing, a sensing-assisted Kalman filter-based approach for channel state information (CSI) estimation is proposed in [26], which improves CSI accuracy through angle-of-arrival (AoA) estimation. For MIMO-OFDM ISAC systems, an angle-subspace-based CSI refinement and angle-domain optimization scheme for angle-of-departure (AoD) estimation is introduced in [27]. To enhance uplink communication performance by leveraging sensing parameters, the work in [28] formulates CE and SD as a constrained bilinear recovery problem and develops a bilinear unitary approximate message passing algorithm to solve it. For orthogonal time–frequency space modulation, a sparse Bayesian learning algorithm is proposed to improve CE accuracy by exploiting the joint sparsity between sensing and communication channels [29]. To reduce beam training overhead and improve communication throughput, reference signals are utilized in [30] for sensing-assisted beam management, simultaneously accelerating beam failure detection and recovery procedures. Furthermore, to fully exploit the rich echo information in wireless environments, a communication echo sensing-assisted CE method is proposed in [31]. These methods provide valuable insights for CE in UAV-assisted OFDM systems. However, they have not adequately incorporated the dominant LoS characteristics inherent in UAV-assisted communication scenarios.

CA technology represents a promising solution for enhancing data transmission bandwidth in UAV-assisted OFDM systems. Several studies have investigated CE methods in CA-enabled wireless communication systems [32,33,34]. Specifically, the time–frequency channel correlation across different component carriers (CCs) in OFDM systems is exploited to improve CE accuracy [32]. In [33], a subchannel selection strategy for CA-OFDM systems is developed based on the analysis of CE error. In UAV-assisted CA-OFDM systems, employing adaptive pilot interval and power allocation for CE achieves both performance improvement and reduced pilot overhead [34]. In [35], a CA experimental testbed is developed to evaluate the CE performance of spectrum-efficient frequency division multiplexing and OFDM systems. Furthermore, an interleaved pilot structure based on CA is proposed in [36] for enhanced ISAC signal design and sensing performance, demonstrating its advantages in CA-enabled ISAC systems. Incorporating CA technology effectively meets the high-bandwidth requirements in UAV-assisted OFDM systems, thereby enabling applications such as real-time video transmission, multi-UAV cooperative operations, and complex environmental sensing.

### 1.3. Motivation and Contributions

The summary of the related works is provided in Table 1. As previously discussed, CE in UAV-assisted OFDM systems still faces challenges, such as inadequate exploitation of channel characteristics, underutilization of sensing information, and inability to meet high-bandwidth demands. To address these issues, this paper proposes an LoS and echo sensing-aided CE method for UAV-assisted OFDM systems with CA technology. The contributions of this paper are summarized as follows:

1. We propose a prior information extraction method by leveraging LoS and echo sensing. Specifically, radar signal processing techniques are employed to analyze echo signals for dynamically capturing the environmental channel characteristics. Correspondingly, LoS sensing is utilized to acquire the dominant path characteristics inherent to UAV scenarios. By fusing the two sensing modalities, the subsequent CE is supplied with richer priors that capture both dynamic environmental echoes and quasi-static path information.
2. Based on the extracted LoS and echo sensing prior information, we propose an LoS and echo sensing-aided CE method for CA-enabled UAV-assisted OFDM systems. This method actively suppresses noise and false path interference by using the environmental prior information from echo sensing. Furthermore, it employs the sensed LoS component as a reference to calibrate the path detection threshold. By jointly leveraging these two sensing modalities, an adaptive path detection threshold is developed to enhance CE accuracy.
3. By leveraging the LoS path characteristics, we establish a novel channel reconstruction paradigm for CA systems, termed path sharing. The core of this paradigm lies in identifying and exploiting the underutilized structural correlation between the PCC and SCC channels. Unlike conventional independent SCC estimation, the proposed method reuses the PCC channel state to reconstruct the LoS paths for the SCCs, which is beneficial for reducing pilot overhead. Furthermore, this path-sharing mechanism is systematically extended to NLoS paths, forming a three-stage channel reconstruction framework. The proposed method fundamentally breaks the linear scaling of pilot overhead with the number of carriers, thereby paving the way for enhancing the spectral efficiency of CA systems.

### 1.4. Outline and Notation

The remainder of this paper is organized as follows: Section 2 introduces the signal model for CA communications and the system model for echo sensing. Section 3 elaborates on the proposed LoS and echo sensing-assisted CA–CE method. The numerical results are presented in Section 4. Finally, Section 5 concludes the paper. Table 2 shows the abbreviations and descriptions used in this paper.

*Notations*: Boldface lowercase and uppercase letters represent the vector and matrix, respectively. ${\left(\;\cdot \;\right)}^{H}$ stands for the conjugate transpose. ∗ denotes linear convolution. $E\left(\;\cdot \;\right)$ denotes the complex expectation operation. $\delta \left(\;\cdot \;\right)$ represents the Dirac delta function. The circularly symmetric Gaussian distribution with mean ω and variance $\sigma^{2}$ is denoted by $\mathrm{CN}(\omega ,\sigma^{2})$, and ∼ stands for “distributed as”. ${∥\;\cdot \;∥}_{2}^{2}$ is the Euclidean norm. $F_{N}$ is the *N*-point normalized N×N discrete Fourier transform (DFT) matrix. $\left\lceil x\right\rceil$ denotes the ceiling operation on *x*. $C^{M\times N}$ denotes M×N dimensional complex matrices. ∩ denotes the intersection set.

## 2. System Model

This section presents the system model utilized in this paper. Specifically, Section 2.1 introduces the communication model based on CA-OFDM, while Section 2.2 describes the echo sensing model.

### 2.1. CA-OFDM Communication Model

**Figure 1:**
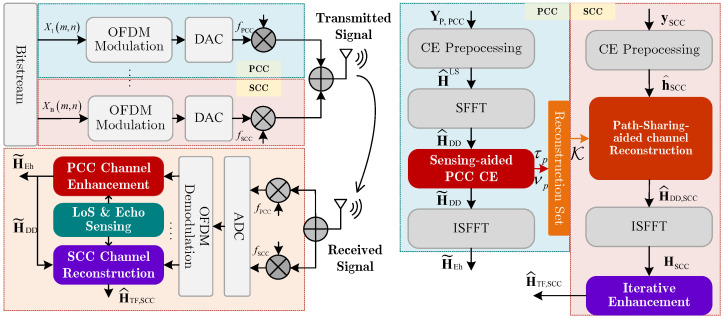
System model with processing flowchart.

As illustrated in Figure 1, the UAV-assisted OFDM system with CA is considered in this paper, which employs *B* CCs and *M* subcarriers. At the transmitter, an inverse discrete Fourier transform (IDFT) is applied to transform the data/pilot symbol $X_{b}\left(m\right)$ (with b=1,2,…,B) on the *m*-th subcarrier (with m=0,1,…,M−1) of the *b*-th CC into the time domain $x_{b}\left(n\right)$ (with n=0,1,…,M−1), which is expressed as [32,37]

(1) $$x_{b}\left(n\right)=\frac{1}{M}\sum_{m=0}^{M-1}X_{b}\left(m\right)\exp\left(j2\pi \frac{nm}{M}\right).$$

According to [16,32], after appending a cyclic prefix (CP), the time-domain transmitted signal $x_{b,\mathrm{CP}}\left(n\right)$ of the *b*-th CC is given by

(2) $$x_{b,\mathrm{CP}}\left(n\right)=\left\{\begin{array}{cc} x_{b}\left(n+N\right), & n=-L_{\mathrm{CP}},\ldots ,-1\\ x_{b}\left(n\right), & n=0,1,\ldots ,N-1 \end{array}\right.,$$

where $L_{\mathrm{CP}}$ denotes the length of CP. At the receiver, the received signal $y_{b,\mathrm{CP}}\left(n\right)$ of the *b*-th CC is expressed as [32]

(3) $$y_{b,\mathrm{CP}}\left(n\right)=x_{b,\mathrm{CP}}\left(n\right)*h_{b}\left(n\right)+w_{b}\left(n\right),$$

where $h_{b}\left(n\right)$ represents the channel impulse response (CIR) of the *b*-th CC, and $w_{b}\left(n\right)$ denotes additive white Gaussian noise (AWGN) with zero mean and variance $\sigma_{w}^{2}$. According to [16], the CIR of the *b*-th CC, denoted as $h_{b}\left(n\right)$, is given by (To simplify the complexity of the research, this paper adopts the tap delay line (TDL) model in [16] as an example to characterize the UAV channels. Other air-to-ground channel models can also be applied).

(4) $$h_{b}\left(n\right)=\underset{≜h_{\mathrm{LoS}}}{\underset{︸}{\sqrt{\frac{K}{K+1}}h_{0,b}\delta \left(n-\tau_{0,b}\right)}}+\underset{≜h_{\mathrm{NLoS}}}{\underset{︸}{\sqrt{\frac{1}{K+1}}\sum_{l=1}^{L-1}h_{l,b}\delta \left(n-\tau_{l,b}\right)}},$$

where K denotes the Rician factor; $h_{0,b}$ and $\tau_{0,b}$ represent the channel gain and path delay of the LoS component for the *b*-th CC, respectively. $h_{l,b}$ and $\tau_{l,b}$ correspond to the channel gain and path delay of the NLoS components for the *b*-th CC, respectively. δ(·) denotes the Dirac delta function. In this paper, the time-varying fading coefficient $h_{l,b}$ is modeled as a zero-mean Gaussian process following the Jakes Doppler spectrum with independent transmission paths [30]. The channel gains and path delays of different CCs (i.e., $h_{l,b}$ and $\tau_{l,b}$ with different values of *b*) are correlated because they share a common physical environment [32]. However, this correlation becomes weaker as the difference between the carriers increases [32].

Without loss of generality, the length of the CP $L_{\mathrm{CP}}$ is chosen to be larger than that of the CIR to prevent inter-symbol interference (ISI); i.e., $L_{\mathrm{CP}}>L$ [16,32]. At the transmitter, the transmitted wireless data frame consists of *D* OFDM symbols. After removing the CP and applying the discrete Fourier transform (DFT), the equivalent baseband signal $Y_{b}\left(m,d\right)$ on the *m*-th subcarrier of the *d*-th symbol (with d=0,1,…,D−1) for the *b*-th CC at the gBS receiver is expressed as [31,37,38,39]

(5) $$Y_{b}\left(m,d\right)=H_{b}\left(m,d\right)X_{b}\left(m,d\right)+W_{b}\left(m,d\right),$$

where $H_{b}\left(m,d\right)$, $X_{b}\left(m,d\right)$, and $W_{b}\left(m,d\right)$ denote the channel frequency response (CFR), the transmitted data/pilot symbol, and the AWGN term with zero mean and variance $\sigma_{w}^{2}$, respectively, for the *m*-th subcarrier of the *d*-th symbol for the *b*-th CC. Based on the received signal $Y_{b}\left(m,d\right)$, the initial CFR is obtained using a classical CE method, such as the least squares (LS) approach. Subsequently, sensing-aided prior information is utilized to refine or reconstruct the initial CFR.

### 2.2. Echo Sensing Model

In next-generation wireless communication systems, echo signals, previously regarded as interference or noise, are now recognized as a valuable source of sensing information and have become one of the key enablers for ISAC designs [31,40]. To this end, this paper explores the extraction of sensing information from echo signals at the gBS.

The transmitted signal comprises *K* OFDM symbols, each containing *S* subcarriers. At the gBS transmitter, after performing the IDFT operation and CP insertion, the transmitted signal in the time domain, denoted as $x_{\mathrm{Sen}}\left(t\right)$, is expressed as [41,42,43]

(6) $$x_{\mathrm{Sen}}\left(t\right)=\sum_{k=0}^{K-1}\sum_{s=0}^{S-1}X_{\mathrm{Sen}}\left(k,s\right)e^{j2\pi s\Delta ft}g\left(t-kT_{o}\right),$$

where $X_{\mathrm{Sen}}\left(k,s\right)$ with k=0,1,…,K−1, and s=0,1,…,S−1 denotes the transmitted symbol on the *s*-th subcarrier of the *k*-th OFDM symbol, Δf represents the subcarrier spacing, $T_{o}$ denotes the duration of an OFDM symbol (including the CP duration $T_{c}$ and the symbol duration $T_{d}$), and g(·) represents the rectangular pulse function. To prevent ISI, the duration of the CP, denoted as $T_{c}$, is configured to be longer than both the maximum multipath delay and the delay corresponding to the maximum detectable range [31,41]. At the gBS receiver, the echo signal $y_{\mathrm{Sen}}\left(t\right)$ received through *P* propagation paths is expressed as [31,41]

(7) $$y_{\mathrm{Sen}}\left(t\right)=\sum_{p=1}^{P}\sum_{k=0}^{K-1}\sum_{s=0}^{S-1}\alpha_{p}X_{\mathrm{Sen}}\left(k,s\right)e^{j2\pi s\Delta f\left(t-\tau_{p}\right)}e^{j2\pi f_{D,p}t}g\left(t-kT_{o}-\tau_{p}\right)+w\left(t\right),$$

where $\alpha_{p}$, $\tau_{p}$, and $f_{D,p}$ denote the amplitude, delay, and Doppler shift of the $p\left(p=1,2,\ldots ,P\right)$-th path, respectively, and $w\left(t\right)$ represents the AWGN with zero mean and variance $\sigma_{w}^{2}$. After CP removal, the received echo signal $y_{\mathrm{Sen}}\left(t\right)$ is transformed into the frequency domain, denoted as $Y_{\mathrm{Sen}}\left(k,s\right)$, yielding [41,42]

(8) $$Y_{\mathrm{Sen}}\left(k,s\right)=\sum_{p=1}^{P}\alpha_{p}X_{\mathrm{Sen}}\left(k,s\right)e^{-j2\pi s\Delta f\tau_{p}}e^{j2\pi kT_{o}f_{D,p}}+W\left(k,s\right),$$

where $W\left(k,s\right)$ represents the AWGN on the *s*-th subcarrier of the *k*-th OFDM symbol. Then, the radar signal processing techniques, such as 2D fast Fourier transform (FFT) [41] and 2D multiple signal classification (MUSIC) [42], are employed to extract sensing information from the received echo signal $Y_{\mathrm{Sen}}\left(k,s\right)$.

## 3. LoS and Echo Sensing-Aided CE

The LoS and echo sensing-aided CE are elaborated on in this section. In Section 3.1, we introduce the LoS sensing method and the extraction of echo sensing-assisted prior information. Based on the extracted LoS and echo sensing information, Section 3.2 elaborates on the process of PCC CE enhancement. By leveraging the LoS path-sharing mechanism between the PCC and SCCs, Section 3.3 reconstructs the SCC channel by utilizing the path information from both PCC and SCCs and further develops a sensing-assisted iteration scheme to enhance the reconstruction accuracy.

### 3.1. Sensing Information Extraction

#### 3.1.1. LoS Sensing

In UAV-assisted OFDM systems, the UAV-to-ground (U2G) link exhibits a high probability of LoS propagation [16,44]. Typically, the LoS path is significantly stronger than individual NLoS paths (by approximately 20 dB) [16]. These observations highlight both the prevalence and detectability of LoS components in U2G links, thus motivating the exploitation of these components to improve CE accuracy. Based on the CIR, the presence of an LoS path is detected using the kurtosis of the received power. By denoting the kurtosis as κ, we have [16]

(9) $$\kappa =\frac{E\left[{\left(\left|h\left(\tau \right)\right|-\mu_{\left|h\left(\tau \right)\right|}\right)}^{4}\right]}{\sigma_{\left|h\left(\tau \right)\right|}^{4}},$$

where τ and $h\left(\tau \right)$ represent the tap delay and CIR of the transmission path, respectively, while $\mu_{\left|h\left(\tau \right)\right|}$ and $\sigma_{\left|h\left(\tau \right)\right|}$ denote the mean and standard deviation of $h\left(\tau \right)$. Due to the notably higher kurtosis value in LoS scenarios compared to NLoS conditions, the presence of an LoS path can be effectively detected when the computed value of κ exceeds a predefined threshold [16]. In this paper, the kurtosis-based detection of the received power is used as an example to demonstrate the feasibility of LoS sensing via received signals. It is noteworthy that other LoS sensing methods are equally applicable, although they are not elaborated on here.

#### 3.1.2. Echo Sensing Information Extraction

By utilizing the received signal $Y_{\mathrm{Sen}}\left(k,s\right)$ from Equation (8) and the known transmitted signal $X_{\mathrm{Sen}}\left(k,s\right)$, the channel transfer function of the echo signal is derived. By denoting the transfer function as $H_{\mathrm{Sen}}\left(k,s\right)$, we have [31]

(10) $$H_{\mathrm{Sen}}\left(k,s\right)=\frac{Y_{\mathrm{Sen}}\left(k,s\right)}{X_{\mathrm{Sen}}\left(k,s\right)}=\sum_{p=1}^{P}\alpha_{p}e^{-j2\pi s\Delta f\tau_{p}}e^{j2\pi kT_{o}f_{D,p}}+\bar{W}\left(k,s\right),$$

where $\bar{W}\left(k,s\right)$ represents AWGN satisfying $\bar{W}\left(k,s\right)∼CN\left(0,\sigma_{\mathrm{Sen}}^{2}\right)$, and $\sigma_{\mathrm{Sen}}^{2}$ denotes the noise variance. Then, the delay-Doppler (DD) periodogram $G_{\mathrm{Sen}}\left(v,q\right)$ is obtained by applying the 2D FFT, which is given by [31]

(11) $$\begin{array}{c} G_{\mathrm{Sen}}\left(v,q\right)=\frac{1}{K}\sum_{k=0}^{K-1}\sum_{s=0}^{S-1}H_{\mathrm{Sen}}\left(k,s\right)e^{j2\pi \left(\frac{s}{S}v-\frac{k}{K}q\right)}\\ \;\;\;\;\;\;\;\;\;\;\;\;\;\;\;\;\approx \frac{1}{K}\sum_{k=0}^{K-1}\sum_{s=0}^{S-1}\sum_{p=1}^{P}\alpha_{p}e^{j2\pi \left(\frac{s}{S}v+kT_{o}f_{D,p}-\frac{k}{K}q-s\Delta f\tau_{p}\right)} \end{array},$$

where v=0,1,…,K−1 and q=0,1,…,S−1. Based on the 2D periodogram, the delay and Doppler shift parameters of resolvable paths are estimated using a 2D cell-averaging constant false alarm rate (CA-CFAR) detector [31]. By representing the set of indices for the reference cells in the reference window as R, the average power $\bar{\beta }$ is then given by [31]

(12) $$\bar{\beta }=\frac{1}{N_{g}}\sum_{\left(v,q\right)\in R}{\left|G_{\mathrm{Sen}}\left(v,q\right)\right|}^{2},$$

where $N_{g}$ denotes the number of reference cells in set R. The detection threshold $T_{h}$ is then constructed as [31]

(13) Th=NgPf−1−1NgNg−1·β¯,

where $P_{f}$ is the predefined false alarm probability for the 2D CA-CFAR detector. Based on the detection threshold $T_{h}$, a hypothesis test is formulated as follows

(14) $${\left|G_{\mathrm{ES}}\left(\hat{v},\hat{q}\right)\right|}^{2}\overset{H_{1}}{\underset{H_{0}}{≷}}T_{h},$$

where the index of the cell under test is denoted by $\left(\hat{v},\hat{q}\right)$, and $H_{1}$ and $H_{0}$ represent the hypotheses of the presence and absence of an echo path, respectively. After detecting all peaks in the DD periodogram, a set I of sensing information is formed. The number of resolvable echo paths, denoted by *G*, is determined by the cardinality of set I; i.e., $G={∥I∥}_{0}$. Accordingly, the sensing information set I indexed by $\left({\hat{v}}_{g},{\hat{q}}_{g}\right)$ with g=1,2,…,G is expressed as

(15) $$I≜{\left\{{\hat{\tau }}_{g}=\frac{{\hat{v}}_{g}}{K\Delta f},{\hat{f}}_{D,g}=\frac{{\hat{q}}_{g}}{ST_{o}}\right\}}_{g=1,2,\ldots ,G},$$

where ${\hat{\tau }}_{g}$ and ${\hat{f}}_{D,g}$ denote the delay and Doppler shift of the echo path indexed by $\left({\hat{v}}_{g},{\hat{q}}_{g}\right)$, respectively. Notably, not all detected echo paths in the sensing-derived information set I correspond to those from the UAV. To focus on the CE enhancement by using echo and LoS sensing, the suppression scheme of false paths is similar to that of [31,45,46,47]. As such, the sensing information set I considered herein is assumed to have undergone false path suppression.

### 3.2. CE Enhancement for PCC

Based on the received signal $Y_{b}\left(m,d\right)$ provided in Equation (5), its time–frequency domain matrix representation $Y_{b}\in C^{M\times D}$ is obtained. By denoting the transmitted pilot signal as $X_{b,P}\in C^{M_{1}\times N_{1}}$, the initial CFR ${\hat{H}}_{b,P}^{\mathrm{LS}}\in C^{M_{1}\times N_{1}}$ is estimated using the classical LS method. Its $\left(m_{1},n_{1}\right)$-th element, denoted as ${\hat{H}}_{b,P}^{\mathrm{LS}}\left(m_{1},n_{1}\right)$, is given by

(16) $${\hat{H}}_{b,P}^{\mathrm{LS}}\left(m_{1},n_{1}\right)=\frac{Y_{b,P}\left(m_{1},n_{1}\right)}{X_{b,P}\left(m_{1},n_{1}\right)},$$

where $Y_{b,P}\left(m_{1},n_{1}\right)$ represents the element at the $m_{1}$-th subcarrier and $n_{1}$-th symbol of the received pilot signal matrix $Y_{P,\;\mathrm{PCC}}\in C^{M_{1}\times N_{1}}$, and $X_{b,P}\left(m_{1},n_{1}\right)$ denotes the element at the $m_{1}$-th subcarrier and $n_{1}$-th symbol of the transmitted pilot signal matrix $X_{P,\;\mathrm{PCC}}\in C^{M_{1}\times N_{1}}$. $M_{1}$ and $N_{1}$ indicate the number of pilots along the subcarrier direction and the OFDM symbol direction, respectively. Subsequently, linear interpolation is employed to obtain the complete channel matrix ${\hat{H}}^{\mathrm{LS}}\in C^{M\times D}$ in the time–frequency domain.

#### 3.2.1. Sensing-Based Prior Information Derivation

Based on the prior information set I (provided in Equation (15)), the prior information of multipath delays and Doppler shifts is obtained. According to [31], the delay spread $\sigma_{\tau }$ is calculated as (This paper leverages the delay spread to design sensing-assisted prior information and can tolerate the presence of some false paths because those within the delay spread do not affect the proposed method).

(17) $$\sigma_{\tau }=\tau_{\max}-\tau_{\min}=\underset{{\hat{\tau }}_{g}}{\arg\max}\left\{{\hat{\tau }}_{g}\right\}-\underset{{\hat{\tau }}_{g}}{\arg\min}\left\{{\hat{\tau }}_{g}\right\},$$

where $\tau_{\min}$ and $\tau_{\max}$ denote the first arrival path delay and the maximum path delay, respectively. Correspondingly, the estimated Doppler spread $\sigma_{\nu }$ is given by

(18) $$\sigma_{\nu }=\underset{{\hat{f}}_{D,g}}{\arg\max}\left\{\left|{\hat{f}}_{D,g}\right|\right\}-\left(-\underset{{\hat{f}}_{D,g}}{\arg\max}\left\{\left|{\hat{f}}_{D,g}\right|\right\}\right)=2\underset{{\hat{f}}_{D,g}}{\arg\max}\left\{\left|{\hat{f}}_{D,g}\right|\right\}.$$

The detailed derivations of delay spread and Doppler spread are provided in Appendix A.

#### 3.2.2. Sensing-Aided CE for PCC

It is noteworthy that the delay spread $\sigma_{\tau }$ and the Doppler spread $\sigma_{\nu }$ exhibit more pronounced characteristics in the DD domain. Therefore, the symplectic finite Fourier transform (SFFT) is employed to transform the initial CFR matrix ${\hat{H}}^{\mathrm{LS}}$ into the DD domain [48]; i.e.,

(19) $${\hat{H}}_{\mathrm{DD}}=F_{M}^{H}{\hat{H}}^{\mathrm{LS}}F_{D},$$

where ${\hat{H}}_{\mathrm{DD}}\in C^{M\times D}$ denotes the initial channel response in the DD domain. When *D* is an odd number, ${\hat{H}}_{\mathrm{DD}}$ is expressed as

(20) H^DD=h^1,−D−1D−122…h^1,−1h^1,0h^1,1…h^1,D−1D−122h^2,−D−1D−122…h^2,−1h^2,0h^2,1…h^2,D−1D−122⋮⋱⋮⋮⋮⋱⋮h^M,−D−1D−122…h^M,−1h^M,0h^M,1…h^M,D−1,D−122,

where ${\hat{h}}_{j,f}$ with j∈[1,M], and f∈[−(D−1)(D−1)22,(D−1)(D−1)22] represents the channel response at the $\left(j,f\right)$-th grid in the DD domain. For the case where *D* is an even number, we have

(21) H^DD=h^1,−DD22…h^1,−1h^1,0h^1,1…h^1,DD22−1h^2,−DD22…h^2,−1h^2,0h^2,1…h^2,DD22−1⋮⋱⋮⋮⋮⋱⋮h^M,−DD22…h^M,−1h^M,0h^M,1…h^M,DD22−1,

where f∈[−DD22,DD22−1]. Due to the sparsity of the channel ${\hat{H}}_{\mathrm{DD}}$, a path detection threshold is established using both LoS sensing and echo-based sensing information to fully exploit the channel sparsity. Consequently, the channel response ${\hat{h}}_{j,f}$ and the estimated delay spread $\sigma_{\tau }$ (provided in Equation (17)) and Doppler spread $\sigma_{\nu }$ (provided in Equation (18)) are utilized for equivalent noise variable ${\hat{\sigma }}^{2}$ estimation, which is expressed as

(22) σ^2=∑j=1M∑f=−(D−1)(D−1)22(D−1)(D−1)22h^j,f2−∑j=1στστTτTτ∑f=−σνσν2Tν2Tνσνσν2Tν2Tνh^j,f2MD−στστTτTτ·σνσνTνTν+1,Disanoddnumber∑j=1M∑f=−DD22DD22−1h^j,f2−∑j=1στστTτTτ∑f=−σνσν2Tν2Tνσνσν2Tν2Tνh^j,f2MD−στστTτTτ·σνσνTνTν+1,   Disanevennumber,

where Tτ=11MΔfComMΔfCom and Tν=ΔfComΔfComDD denote the delay resolution and Doppler resolution, respectively, and $\Delta f_{\mathrm{Com}}$ represents the subcarrier spacing of the communication system. According to [31], the detection threshold for CE enhancement, denoted as ${\tilde{T}}_{h}$, is designed by

(23) $${\tilde{T}}_{h}=\sqrt{-2{\hat{\sigma }}^{2}\ln P_{\mathrm{fa}}},$$

where $P_{\mathrm{fa}}$ denotes the predefined false alarm probability for detecting transmission paths. Employing ${\tilde{T}}_{h}$ effectively mitigates estimation errors, thereby enhancing CE accuracy. However, ${\tilde{T}}_{h}$ fails to fully capitalize on the sensed LoS path advantage, rendering it vulnerable to multipath interference and degrading the performance of resolvable path detection. To address this limitation, the sensed LoS path is further incorporated to refine the threshold, leading to an enhanced LoS-assisted threshold $T_{h,\mathrm{CE}}$ defined as

(24) $$T_{h,\mathrm{CE}}=\varepsilon {\tilde{T}}_{h}\left(1+\delta_{\mathrm{LoS}}\right),$$

where ε denotes the threshold factor. According to [49], the threshold factor ε is expressed as ε=PfL−1−1β−1β−1−1, where $P_{\mathrm{fL}}$ and β=στστTτTτ·σνσνTνTν+1 represent the false alarm probability based on the LoS threshold and the size of the reference window, respectively. $\delta_{\mathrm{LoS}}$ represents the path factor associated with the sensed LoS path. For the estimated DD domain channel response, the path factor $\delta_{\mathrm{LoS}}$ is given by

(25) δLoS=hLoS∑j=1στστTτTτ∑f=−σνσν2Tν2Tνσνσν2Tν2Tνh^j,f2+δnoise2,

where $h_{\mathrm{LoS}}$ denotes the complex gain of the LoS path and $\delta_{\mathrm{noise}}^{2}$ represents the noise factor. Despite the presence of the LoS path, a significant presence of scattered components indicates the existence of NLoS paths in addition to the inevitable estimation noise at the gBS receiver. Thus, the noise factor $\delta_{\mathrm{noise}}^{2}$ is defined as

(26) δnoise2=∑j=1M∑f=−(D−1)(D−1)22(D−1)(D−1)22h^j,f2−∑j=1στστTτTτ∑f=−σνσν2Tν2Tνσνσν2Tν2Tνh^j,f2∑j=1M∑f=−(D−1)(D−1)22(D−1)(D−1)22h^j,f2,Disanoddnumber∑j=1M∑f=−DD22DD22−1h^j,f2−∑j=1στστTτTτ∑f=−σνσν2Tν2Tνσνσν2Tν2Tνh^j,f2∑j=1M∑f=−(D−1)(D−1)22(D−1)(D−1)22h^j,f2,Disanevennumber.

By representing the enhanced channel matrix as ${\bar{H}}_{\mathrm{DD}}\in C^{M\times D}$, its (j,f)-th element is determined by

(27) $${\bar{h}}_{j,f}=\left\{\begin{array}{c} {\hat{h}}_{j,f},\;\;\;\left|{\hat{h}}_{j,f}\right|\geq T_{h,\mathrm{CE}}\\ 0,\;\;\;\;\;\;\;\;\mathrm{otherwise} \end{array}\right.,$$

where ${\bar{h}}_{j,f}$ denotes the $\left(j,f\right)$-th element of ${\bar{H}}_{\mathrm{DD}}$. In the absence of an LoS path, ${\tilde{T}}_{h}$ (in Equation (23)) is utilized as the detection threshold, thereby ensuring the applicability of the proposed method. Based on Equation (22), the delay spread $\sigma_{\tau }$ and Doppler spread $\sigma_{\nu }$ are leveraged as constraints on the channel response to further suppress estimation errors. By denoting the enhanced channel matrix of ${\bar{H}}_{\mathrm{DD}}$ as ${\tilde{H}}_{\mathrm{DD}}\in C^{M\times D}$, its $\left(j,f\right)$-th entry, i.e., ${\tilde{h}}_{j,f}$, is given by

(28) $${\tilde{h}}_{j,f}=\left\{\begin{array}{cc} {\bar{h}}_{j,f}, & 1\leq j\leq \left\lceil \frac{\sigma_{\tau }}{T_{\tau }}\right\rceil ,\left|f\right|\leq \left\lceil \frac{\sigma_{\nu }}{2T_{\nu }}\right\rceil \\ 0, & \mathrm{otherwise} \end{array}\right..$$

Subsequently, the channel matrix ${\tilde{H}}_{\mathrm{DD}}$ in the DD domain is transformed into the time–frequency domain by using the inverse symplectic finite Fourier transform (ISFFT) [48], yielding the enhanced time–frequency channel matrix ${\tilde{H}}_{\mathrm{Eh}}\in C^{M\times D}$; i.e.,

(29) $${\tilde{H}}_{\mathrm{Eh}}=F_{M}{\tilde{H}}_{\mathrm{DD}}F_{D}^{H}.$$

Thus, by leveraging the sensing-derived prior information constructed from both LoS and echo sensing, the proposed method significantly enhances the CE performance. The implementation details of the sensing-aided CE enhancement for PCC are summarized in Algorithm 1.
**Algorithm 1** Sensing-Aided CE Enhancement for PCC**Input:** The received pilot signal $Y_{P,\;\mathrm{PCC}}$, the transmitted pilot signal $X_{P,\;\mathrm{PCC}}$, the prior information set I, the delay resolution $T_{\tau }$ and Doppler resolution $T_{\nu }$, the predefined false alarm probability $P_{\mathrm{fa}}$.**Output:** The enhanced channel of PCC ${\tilde{H}}_{\mathrm{Eh}}$.  1:**Initial CFR extraction:**  2:Estimate initial CFR of PCC ${\hat{H}}_{b,P}^{\mathrm{LS}}$ using Equation (16);  3:Acquire the DD channel matrix ${\hat{H}}_{\mathrm{DD}}$ through Equation (19);  4:**Sensing-based Prior Information Derivation:**  5:Calculate the estimated delay spread $\sigma_{\tau }$ via Equation (17);  6:Obtain the estimated Doppler spread $\sigma_{\nu }$ using Equation (18);  7:Estimate the equivalent noise variable ${\hat{\sigma }}^{2}$ using Equation (22);  8:Calculate the channel path detection threshold ${\tilde{T}}_{h}$ through Equation (23);  9:Obtain the enhanced LoS-assisted threshold $T_{h,\mathrm{CE}}$ via Equation (24);10:**PCC channel enhancement processing:**11:**Stage 1:** The path detection threshold-based channel enhancement:12:**for **j=1,2,…,M**do**13:       **for** f=−(D−1)(D−1)22,…,(D−1)(D−1)22 (or −DD22,…,DD22−1) **do**14:           **if** $\left|{\hat{h}}_{j,f}\right|\geq T_{h,\mathrm{CE}}$ **then**15:                 ${\bar{h}}_{j,f}={\hat{h}}_{j,f}$;16:           **else**17:              ${\bar{h}}_{j,f}=0$;18:           **end if**19:       **end for**20:**end for**21: **Stage 2:** The Delay and Doppler Spread-based channel enhancement:22:**for** j=1,2,…,M**do**23:    **for** f=−(D−1)(D−1)22,…,(D−1)(D−1)22 (or −DD22,…,DD22−1) **do**24:         **if** $1\leq j\leq \left\lceil \frac{\sigma_{\tau }}{T_{\tau }}\right\rceil \&\&\left|f\right|\leq \left\lceil \frac{\sigma_{\nu }}{2T_{\nu }}\right\rceil$ **then**25:              ${\tilde{h}}_{j,f}={\bar{h}}_{j,f}$;26:        **else**27:               ${\tilde{h}}_{j,f}=0$;28:        **end if**29:    **end for**30:**end for**31:Obtain the enhanced CE ${\tilde{H}}_{\mathrm{Eh}}$ using Equation (29).

### 3.3. Sensing and Path-Sharing-Aided Channel Reconstruction for SCCs

By setting the detection threshold $T_{h,\mathrm{CE}}$, the channel path information of the PCC in the DD domain is achieved and thus forms a reconstructed set of path delays and Doppler shifts K as

(30) $$K≜{\left\{\tau_{p},\nu_{p}\right\}}_{p=1,2,\ldots ,P},$$

where $\tau_{p}$ and $\nu_{p}$ denote the delay and Doppler shift of the *p*-th resolvable path, respectively. By leveraging the reconstructed set K, an LoS-inspired CE scheme (based on path-sharing between PCC and SCCs) is proposed to reconstruct the channels of SCCs. Without loss of generality, an arbitrary frequency band is used as an example to demonstrate the channel reconstruction stage for SCCs. It should be noted that the proposed scheme is applicable to multiple frequency bands. The implementation details of the SCC channel reconstruction scheme assisted by sensing and path-sharing are provided in Algorithm 2, and the detailed derivation is as follows.
**Algorithm 2** Sensing and Path-Sharing-Aided Channel Reconstruction for SCCs**Input:** The reconstruction-set of path delays and Doppler shifts K, the received pilot vector $y_{\mathrm{SCC}}$, the transmitted pilot vector $p_{\mathrm{SCC}}$, number of iterations $N_{\mathrm{iter}}$.**Output:** The reconstruction channel of SCells ${\hat{H}}_{\mathrm{SCell}}$.  1:Initial SCells channel ${\hat{H}}_{\mathrm{DD},\mathrm{SCells}}=0_{M\times D}$;  2:**Stage 1: LoS Path-Based Reconstruction:**  3:Obtain the Doppler index of the LoS path of SCC via Equations (31) and (32);  4:Estimate the channel path gain through Equations (33) and (34);  5:Reconstruct LoS channel path for SCC using Equation (35);  6:**Stage 2: NLoS Path-Based Reconstruction:**  7:Obtain the shared NLoS path between the PCC and SCC through Equation (36);  8:**if** A∩B≠∅**then**  9:    **for** g=1,2,…,G **do**10:          ${\hat{\nu }}_{g}=\;\left\lceil \frac{\nu_{g}T_{\nu ,\mathrm{PCC}}f_{\mathrm{SCC}}}{T_{\nu ,\mathrm{SCC}}f_{\mathrm{PCC}}}\right\rceil$;11:          ${\hat{h}}_{\mathrm{DD},\mathrm{SCC}}^{{\hat{\tau }}_{g},{\hat{\nu }}_{g}}={\hat{h}}_{\mathrm{SCC}}\left({\hat{\tau }}_{g}\right)$;12:    **end for**13:**else**14:    break;15:**end if**16:**Stage 3: Iterative Channel Reconstruction and Enhancement:**17:**for** $n_{\mathrm{iter}}=1,2,\ldots ,N_{\mathrm{iter}}$**do**18:    Obtain the CFR $H_{\mathrm{SCC}}$ of SCells using Equation (40);19:    Employ the zero forcing (ZF) equalization via Equation (41);20:    Demodulate and remodulate the equalization symbol to obtain $X_{\mathrm{SCC},\mathrm{data},n_{iter}}$;21:     Obtain the complete time–frequency signal $X_{\mathrm{SCC},n_{iter}}$ through Equation (42);22:    Regard $X_{\mathrm{SCC},n_{iter}}$ as pilot to estimate the SCC channel through Equation (16);23:     Eliminate reconstruction errors via Equations (19)–(28);24:**end for**25:Obtain the enhancement SCC channel ${\tilde{H}}_{\mathrm{TF},\mathrm{SCC}}$;

#### 3.3.1. LoS Path-Based Reconstruction

In the presence of an LoS path, the transmission delays of the PCC and SCCs are approximately equal; i.e., ${\hat{\tau }}_{\mathrm{LoS},\mathrm{PCC}}={\hat{\tau }}_{\mathrm{LoS},\mathrm{SCCs}}$. However, the different carrier frequencies of PCC and SCCs result in distinct Doppler shifts. Inspired by [36], the shared paths exhibit correlation under different CCs, which motivates us to utilize this sharing and correlation for SCC channel reconstruction. By respectively denoting the frequencies of PCC and SCC as $f_{\mathrm{PCC}}$ and $f_{\mathrm{SCC}}$, the corresponding Doppler indices are expressed as

(31) $$\left\{\begin{array}{c} \nu_{\mathrm{LoS}}=\frac{f_{d,p}}{T_{\nu ,\mathrm{PCC}}}=\frac{v_{\mathrm{PCC}}f_{\mathrm{PCC}}}{cT_{\nu ,\mathrm{PCC}}}\\ {\hat{\nu }}_{\mathrm{LoS}}=\frac{f_{d,q}}{T_{\nu ,\mathrm{SCC}}}=\frac{v_{\mathrm{SCC}}f_{\mathrm{SCC}}}{cT_{\nu ,\mathrm{SCC}}} \end{array}\right.,$$

where $f_{d,p}$, $v_{\mathrm{PCC}}$, and $T_{\nu ,\mathrm{PCC}}$ denote the Doppler shift of the LoS path, the velocity, and the Doppler resolution of the PCC, respectively. Accordingly, $f_{d,q}$, $v_{\mathrm{SCC}}$, and $T_{\nu ,\mathrm{SCC}}$ represent the Doppler shift of the LoS path, the equivalent velocity, and the Doppler resolution for the SCC, respectively. Due to $v_{\mathrm{PCC}}=v_{\mathrm{SCC}}$, the Doppler index of the LoS path in the SCC satisfies

(32) $${\hat{\nu }}_{\mathrm{LoS}}=\;\left\lceil \frac{\nu_{\mathrm{LoS}}T_{\nu ,\mathrm{PCC}}f_{\mathrm{SCC}}}{T_{\nu ,\mathrm{SCC}}f_{\mathrm{PCC}}}\right\rceil .$$

Based on Equation (32), the Doppler index corresponding to the channel response of the SCC in the DD domain is obtained. However, this shared property does not extend to the complex gain of the channel, i.e., the amplitude and phase of the channel response. Therefore, further estimation of the complex channel gain is required to reconstruct the SCC channel.

To reduce the pilot overhead for the SCC and leverage the channel path-sharing property, a small number of pilot symbols are employed to estimate the complex channel gain. In this paper, the first OFDM symbol in the received frame of the SCC is utilized as the pilot symbol for complex channel gain estimation. By denoting the estimated CFR vector as ${\hat{H}}_{\mathrm{SCC}}\in C^{M\times 1}$, its *m*-th entry, i.e., ${\hat{H}}_{\mathrm{SCC}}\left(m\right)$, is given by

(33) $${\hat{H}}_{\mathrm{SCC}}\left(m\right)=\frac{y_{\mathrm{SCC}}\left(m\right)}{p_{\mathrm{SCC}}\left(m\right)},$$

where $y_{\mathrm{SCC}}\left(m\right)$ and $p_{\mathrm{SCC}}\left(m\right)$ denote the *m*-th entries of the received pilot vector $y_{\mathrm{SCC}}\in C^{M\times 1}$ and the transmitted pilot vector $p_{\mathrm{SCC}}\in C^{M\times 1}$ for the SCC, respectively. By applying the IDFT, the CFR matrix is transformed into the time domain, yielding

(34) $${\hat{h}}_{\mathrm{SCC}}=F_{M}{\hat{H}}_{\mathrm{SCC}},$$

where ${\hat{h}}_{\mathrm{SCC}}\in C^{M\times 1}$ represents the estimated CIR matrix of the SCC. In this paper, the time-domain channel gain ${\hat{h}}_{\mathrm{SCC}}\left(m\right)$ is utilized to approximate the complex channel gain at the corresponding delay in the DD domain. By representing the channel matrix of the SCC in the DD domain as ${\hat{H}}_{\mathrm{DD},\mathrm{SCC}}\in C^{M\times D}$, the channel response corresponding to the index $\left({\hat{\tau }}_{\mathrm{LoS},\mathrm{SCC}},{\hat{\nu }}_{\mathrm{LoS}}\right)$ of the LoS path of the SCC in the DD domain, denoted as ${\hat{h}}_{\mathrm{DD},\mathrm{SCC}}^{\tau_{\mathrm{LoS},\mathrm{SCC}},{\hat{\nu }}_{\mathrm{LoS}}}$, is given by

(35) $${\hat{h}}_{\mathrm{DD},\mathrm{SCC}}^{\tau_{\mathrm{LoS},\mathrm{SCC}},{\hat{\nu }}_{\mathrm{LoS}}}={\hat{h}}_{\mathrm{SCC}}\left({\hat{\tau }}_{\mathrm{LoS},\mathrm{SCC}}\right),$$

where ${\hat{h}}_{\mathrm{SCC}}\left({\hat{\tau }}_{\mathrm{LoS},\mathrm{SCC}}\right)$ denotes the channel response of the LoS path of ${\hat{h}}_{\mathrm{SCC}}$.

#### 3.3.2. NLoS Path-Based Reconstruction

Building upon the LoS path-sharing mechanism, we further extend this path-sharing framework to NLoS scenarios. For clarity, the path delay sets of the PCC and the SCC are defined as $A≜{\left\{\tau_{p}\right\}}_{p=1,\ldots ,P}$ and $B≜{\left\{{\hat{\tau }}_{m}\right\}}_{m=1,\ldots ,M}$, respectively. For the case where the path delay of the PCC coincides with that of the SCC, the intersection set is given by

(36) $$A\cap B=\left\{{\hat{\tau }}_{g},g=1,2,\ldots ,G\right\},$$

where ${\hat{\tau }}_{g}$ denotes the *g*-th shared path with identical delay between the PCC and SCC. By leveraging the proportional relationship between the carrier frequencies of the shared paths, the channel parameters of the SCC in the Doppler direction are reconstructed. According to Equation (32), the Doppler index of the *g*-th path in the SCC is given by

(37) $${\hat{\nu }}_{g}=\;\left\lceil \frac{\nu_{g}T_{\nu ,\mathrm{PCC}}f_{\mathrm{SCC}}}{T_{\nu ,\mathrm{SCC}}f_{\mathrm{PCC}}}\right\rceil .$$

Subsequently, the channel response corresponding to the index of the *g*-th path (i.e., $\left({\hat{\tau }}_{g},{\hat{\nu }}_{g}\right)$) in the DD domain for the SCC, denoted as ${\hat{h}}_{\mathrm{DD},\mathrm{SCC}}^{{\hat{\tau }}_{g},{\hat{\nu }}_{g}}$, is given by

(38) $${\hat{h}}_{\mathrm{DD},\mathrm{SCC}}^{{\hat{\tau }}_{g},{\hat{\nu }}_{g}}={\hat{h}}_{\mathrm{SCC}}\left({\hat{\tau }}_{g}\right),$$

where ${\hat{h}}_{\mathrm{SCC}}\left({\hat{\tau }}_{g}\right)$ denotes the channel response of the *g*-th path of ${\hat{h}}_{\mathrm{SCC}}$. By combining Equation (35) and Equation (38), the $\left(j,f\right)$-th element of the reconstructed channel matrix ${\hat{H}}_{\mathrm{DD},\mathrm{SCC}}\in C^{M\times D}$ of the SCC, denoted as ${\hat{h}}_{\mathrm{DD},\mathrm{SCC}}^{j,f}$, is rewritten as

(39) $$\left\{\begin{array}{c} {\hat{h}}_{\mathrm{DD},\mathrm{SCC}}^{\tau_{\mathrm{LoS},\mathrm{SCC}},{\hat{\nu }}_{\mathrm{LoS}}}={\hat{h}}_{\mathrm{SCC}}\left({\hat{\tau }}_{\mathrm{LoS},\mathrm{SCC}}\right),{\hat{\tau }}_{\mathrm{LoS},\mathrm{PCC}}={\hat{\tau }}_{\mathrm{LoS},\mathrm{SCC}}\\ \begin{array}{c} {\hat{h}}_{\mathrm{DD},\mathrm{SCC}}^{{\hat{\tau }}_{g},{\hat{\nu }}_{g}}={\tilde{h}}_{\mathrm{SCC}}\left({\hat{\tau }}_{g}\right),{\hat{\tau }}_{g}\in \left(A\cap B\right)\\ {\hat{h}}_{\mathrm{DD},\mathrm{SCC}}^{j,f}=0,\mathrm{others} \end{array} \end{array}\right..$$

Thus, an initial reconstruction of the SCC channel is performed by leveraging the channel path-sharing mechanism. In [12,32,36], the channel path-sharing mechanism has been leveraged for joint design among different CCs with different frequency bands. However, the channel paths are not fully shared between the PCC and SCCs, limiting the accuracy of the channel reconstruction. To address this, an iterative processing scheme is employed to reconstruct the non-shared channel paths while mitigating the reconstruction errors.

#### 3.3.3. Iterative Channel Reconstruction and Enhancement for SCCs

With the reconstructed channel ${\hat{H}}_{\mathrm{DD},\mathrm{SCC}}$ (according to Equation (39)), it is transformed into the time–frequency domain by using the ISFFT. By denoting the transformed version as $H_{\mathrm{SCC}}\in C^{M\times D}$, we have

(40) $$H_{\mathrm{SCC}}=F_{M}{\hat{H}}_{\mathrm{DD},\mathrm{SCC}}F_{D}^{H}.$$

By denoting the received signal of the SCC as $Y_{\mathrm{SCC}}\in C^{M\times D}$, the received signal at data positions (denoted as $Y_{\mathrm{SCC},\mathrm{data}}\in C^{M\times \left(D-1\right)}$) and the channel matrix (i.e., $H_{\mathrm{SCC},\mathrm{data}}\in C^{M\times \left(D-1\right)}$) are extracted to perform zero-forcing (ZF) equalization. The $\left(m,d\right)$-th element of the equalized data ${\hat{X}}_{\mathrm{SCC},\mathrm{data}}\in C^{M\times \left(D-1\right)}$, denoted as ${\hat{X}}_{\mathrm{SCC},\mathrm{data}}\left(m,d\right)$, is given by

(41) $${\hat{X}}_{\mathrm{SCC},\mathrm{data}}\left(m,d\right)=\frac{Y_{\mathrm{SCC},\mathrm{data}}\left(m,d\right)}{H_{\mathrm{SCC},\mathrm{data}}\left(m,d\right)},$$

where $Y_{\mathrm{SCC},\mathrm{data}}\left(m,d\right)$ and $H_{\mathrm{SCC},\mathrm{data}}\left(m,d\right)$ denote the $\left(m,d\right)$-th elements of $Y_{\mathrm{SCC},\mathrm{data}}\in C^{M\times \left(D-1\right)}$ and $H_{\mathrm{SCC},\mathrm{data}}\in C^{M\times \left(D-1\right)}$, respectively. Subsequently, data ${\hat{X}}_{\mathrm{SCC},\mathrm{data}}$ is demodulated and then remodulated to obtain $X_{\mathrm{SCC},\mathrm{data}}\in C^{M\times \left(D-1\right)}$. The signal $X_{\mathrm{SCC},\mathrm{data}}$ and the pilot signal $p_{\mathrm{SCC}}$ are concatenated to form the complete time–frequency signal $X_{\mathrm{SCC}}\in C^{M\times D}$; i.e.,

(42) $$X_{\mathrm{SCC}}=\left[p_{\mathrm{SCC}},X_{\mathrm{SCC},\mathrm{data}}\right].$$

Subsequently, $X_{\mathrm{SCC}}$ is utilized as the pilot signal, and the CE for the SCC is performed according to Equation (16), thereby obtaining ${\hat{H}}_{\mathrm{SCC}}\in C^{M\times N}$. Furthermore, the estimated ${\hat{H}}_{\mathrm{SCC}}$ is enhanced using Equation (19) to Equation (29). By leveraging prior information assisted by LoS and echo sensing, the reconstruction errors introduced during the reconstruction stage are mitigated, thereby yielding the enhanced channel matrix ${\hat{H}}_{\mathrm{TF},\mathrm{SCC}}$ for the SCC.

Since the reconstruction errors introduced during the reconstruction stage cannot be eliminated in a single iteration, the operations from Equation (19) to Equation (29) are iteratively applied to enhance the CE accuracy of the SCC, ultimately yielding the enhanced channel matrix ${\tilde{H}}_{\mathrm{TF},\mathrm{SCC}}$ for the SCC.

## 4. Simulation Results and Analysis

In this section, numerical simulations are conducted to validate the effectiveness and robustness of the proposed LoS and echo sensing-assisted CE method in UAV communication scenarios. The simulation parameters and methodology are presented in Section 4.1. In Section 4.2, the computational complexity of the proposed scheme and baseline methods is analyzed. The effectiveness of the proposed method and its robustness against parameter variations are evaluated in Section 4.3 and Section 4.4, respectively. Furthermore, the key results, highlighting their significance and potential application scenarios as well as the observed anomalies or alignments, are presented in Table 3.

### 4.1. Parameter Settings

The basic simulation parameters involved are provided as follows. For the communication system, the number of CCs is set to B=2, with the carrier frequencies of the PCC and SCC being 2.4 GHz and 5.8 GHz, respectively. The duration of each time slot is 1ms. The number of subcarriers and OFDM symbols per slot are set to M=72 and D=14, respectively, with a subcarrier spacing of $\Delta f_{\mathrm{Com}}=15\;\mathrm{kHz}$ and a CP length of LCP=MM44. The PCC employs a comb-type pilot structure with a pilot interval of 2. The false alarm probabilities for the path threshold and the LoS-based threshold are set to $P_{\mathrm{fa}}=10^{-8}$ and $P_{\mathrm{fL}}=10^{-3}$, respectively. The bit stream is modulated by the quadrature phase shift keying (QPSK) mode. For the echo sensing system, an OFDM radar system with a carrier frequency of $f_{c}=2.1$ GHz is considered. The number of subcarriers is S=1024, the number of OFDM symbols is K=256, the subcarrier spacing is Δf=111.25kHz, the OFDM symbol duration is $T_{c}=2.25\;\mu s$, and the total symbol duration (including CP) is $T_{d}=11.25\;\mu s$. According to [31], the false alarm probability of the 2D CA-CFAR detector is set to $P_{f}=10^{-6}$, and the number of reference cells is set to $N_{g}=28$. The power delay profile (PDP) of the channel model adopts the tapped delay line (TDL)-D channel model, and the Rician factor K is randomly generated within the interval $\left[3,13\right]$. Specifically, the TDL-D channel model comprises 13 taps. The first tap is Ricean-distributed with a *K*-factor of 13.3 decibels (dB) at zero delay and 0 dB normalized power, whereas the subsequent 12 taps follow a Rayleigh distribution. These Rayleigh taps have increasing delays (i.e., from 0.035 to 12.525) and progressively decreasing gains (i.e., from −18.8 dB to −30.0 dB) [50]. To account for the non-correlation between different CCs, i.e., the existence of non-shared paths between PCC and SCC channels, their path-number difference (denoted as $\Delta_{L}$) is randomly set as $\Delta_{L}=0$ or $\Delta_{L}=1$. Subsequently, in the parameter robustness analysis, we increase the number of non-shared paths between the PCC and SCC. The UAV velocity and distance are set to v=25m/s and d=500m, respectively. By respectively representing the received signal power and the noise variance as $P_{r}$ and $\sigma_{n}^{2}$, the signal-to-noise ratio (SNR) in dB is defined as $\mathrm{SNR}=10\log_{10}\left(P_{r}/\sigma_{n}^{2}\right)$, and the normalized mean square error (NMSE) is defined as

(43) $$\mathrm{NMSE}=E\left\{\frac{{∥\tilde{H}-H∥}_{2}^{2}}{{∥H∥}_{2}^{2}}\right\},$$

where $\tilde{H}$ is the estimate of the true channel H. To evaluate the effectiveness of the proposed method, the following schemes are adopted in the subsequent simulation analysis:

- LS: Classic least squares with linear interpolation.
- DFT_based: LS with DFT enhancement.
- OMP_based: Classic CS-based CE scheme.
- LoS_based: LoS sensing-based CE enhancement scheme in [16].
- LS_DD: LS enhancement with sensing-aided scheme in DD domain in [31].
- Path_gains_based: Path gain-based CE enhancement scheme in [32].
- ReEsNet: Residual deep CNN-based CE method in [38].
- Channelformer: Transformer-based multi-head attention mechanism CE method in [39].
- Prop_PCC: Proposed CE enhancement scheme for PCC.
- Prop_SCC: Proposed channel reconstruction scheme for SCC.
- Prop_iter: Proposed iterative channel reconstruction and enhancement for SCC.

where “LS”, “DFT_based”, “OMP_based”, “LoS_based”, “LS_DD”, “Path_gains_based”, “ReEsNet”, and “Channelformer” are employed as baseline methods.

### 4.2. Computational Complexity Analysis

Complex multiplication (CM) is employed to evaluate the computational complexity of both the proposed and comparative methods. For “LS”, the CMs are primarily caused by CE at pilot positions and interpolation operations at data positions. Due to the pilot interval of 2, the CMs of “LS” at pilot positions are MDMD22. To obtain the channels at data positions, linear interpolation is applied to recover the complete CFR, requiring additional CMs of MDMD22. Thus, the total CMs of the “LS” are MD. For “DFT_based”, “Path_gains_based”, and “LoS_based”, additional transform-domain operations are required relative to “LS”. This consumes one IDFT and one DFT operation along the subcarrier direction for *D* symbols, resulting in $2M^{2}D$ CMs. Although time-domain filtering or thresholding operations are employed to perform denoising, we ignore these CMs for “DFT_based”, “Path_gains_based”, and “LoS_based” due to their insignificance. Hence, the total CMs for these methods remain approximately $2M^{2}D$. For the CS-based “OMP_based” method, each symbol entails iterative processing with a sparsity of *K*, yielding the CMs of $M^{2}DK$. Additionally, a DFT operation along the subcarrier direction is required to transform the CIR to the frequency domain, with the corresponding CMs of $M^{2}D$. Thus, the total CMs of “OMP_based” are $M^{2}DK+M^{2}D$. For “LS_DD”, the CMs are divided into sensing and communication parts. The sensing part primarily includes a 2D-DFT and a 2D CA-CFAR detection, requiring the CMs of $M^{2}D+MD^{2}$. For CE enhancement processing, the operation involves one IDFT and one DFT, each with additional $M^{2}D+MD^{2}$ CMs relative to “LS”. In total, the CMs for “LS_DD” are $2MD+3M^{2}D+3MD^{2}$. For the DL-based CE enhancement methods in [38,39], the convolutional layers and the fully connected layers primarily contribute to the CMs. Consequently, the CMs of “ReEsNet” and “Channelformer” are $\left(1+\left(4\times 3^{3}+11^{2}\right)\times 16\right)NM$ and $\left(69+5^{2}\times 86+10NM+2N+2M\right)NM$, respectively.

For the proposed method “Prop_PCC”, the CMs for CE are MD. Compared to “LS_DD”, “Prop_PCC” introduces additional sensing operations and LoS-based threshold refinement. Nonetheless, these operations primarily involve additive and subtractive calculations. Hence, the total CMs required for “Prop_PCC” are approximately $2MD+3M^{2}D+3MD^{2}$. For the SCC, the CMs required for complex channel gain estimation are *M*. In addition, one transform-domain operation involving an IDFT and a DFT is needed, with the CMs being $M^{2}D+MD^{2}$. Hence, the total CMs for the “Prop_SCC” are $M+M^{2}D+MD^{2}$. To improve rthe econstruction accuracy of the SCC, the CMs for one iteration of the SCC channel enhancement are $4MD+2M^{2}D+2MD^{2}$. With $N_{\mathrm{iter}}$ iterations, the total CMs of “Prop_iter” are $M+M^{2}D+MD^{2}+N_{\mathrm{iter}}\left(4MD+2M^{2}D+2MD^{2}\right)$.

### 4.3. Effectiveness Analysis

**Figure 2:**
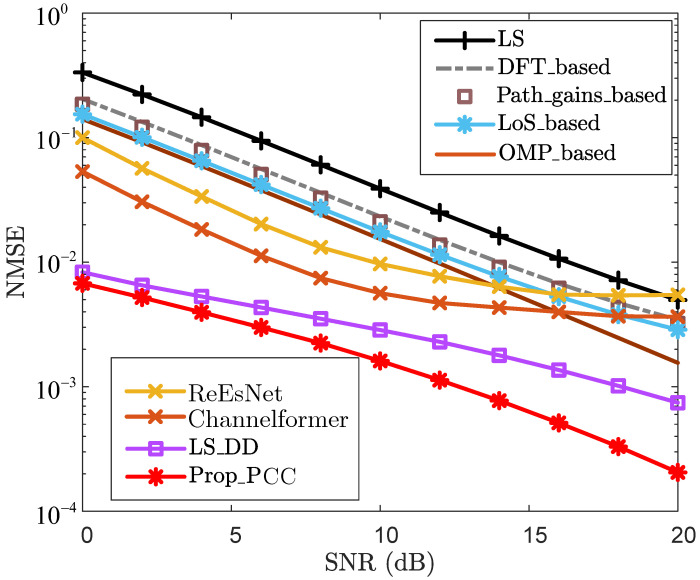
NMSE vs. SNR for PCC.

**Figure 3:**
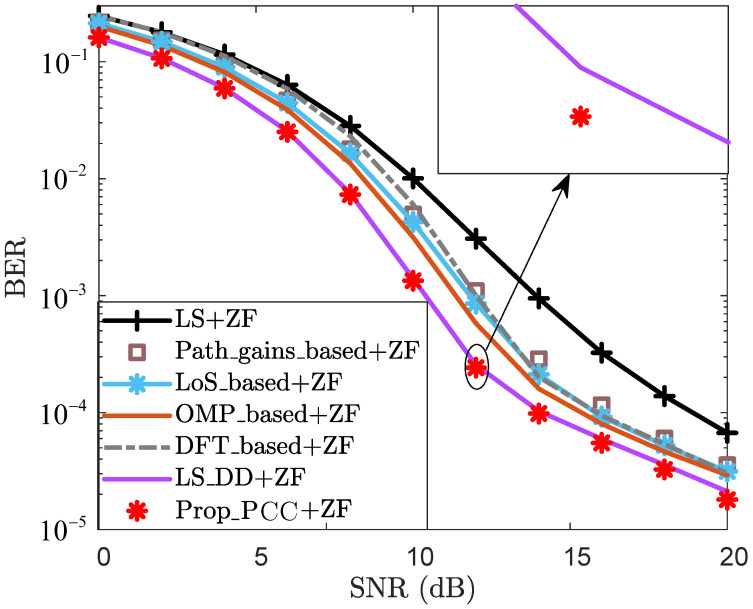
BER vs. SNR for PCC.

To analyze the effectiveness of the proposed method, Figure 2 and Figure 3 present the NMSE and bit-error rate (BER) performance for the PCC, respectively. From Figure 2, for each given SNR, the NMSEs of “DFT_based”, “Path_gains_based”, and “LoS_based” are lower than that of “LS”. For the case where SNR=10dB, the NMSE of “LS” is $3.90\times 10^{-2}$, while the NMSEs of “DFT_based”, “Path_gains_based”, and “LoS_based” are $2.33\times 10^{-2}$, $2.11\times 10^{-2}$, and $1.76\times 10^{-2}$, respectively. Furthermore, “LoS_based” outperforms both “DFT_based” and “Path_gains_based” for each specific SNR in terms of NMSE, demonstrating the effectiveness and superiority of leveraging LoS sensing to enhance CE accuracy. For the CS-based method “OMP_based”, it effectively improves NMSE performance by utilizing sparse reconstruction. However, this method requires precise knowledge of the sparsity, which hinders its practical application. By leveraging echo sensing-aided prior information, “LS_DD” and “Prop_PCC” achieve significantly superior NMSE performance compared to “LS”, “DFT_based”, “Path_gains_based”, “LoS_based”, “OMP_based”, “ReEsNet”, and “Channelformer”. When SNR=12dB, the NMSEs of “LS_DD” and “Prop_PCC” are $2.26\times 10^{-3}$ and $1.13\times 10^{-3}$, respectively. In contrast, the NMSEs of “LS”, “DFT_based”, “Path_gains_based”, “LoS_based”, “OMP_based”, “ReEsNet”, and “Channelformer” all exceed $4.71\times 10^{-3}$. This demonstrates that leveraging echo sensing-aided prior information significantly enhances CE accuracy. Furthermore, “Prop_PCC” consistently outperforms “LS_DD” in terms of NMSE performance for each given SNR. This improvement is attributed to the fact that “Prop_PCC” incorporates LoS sensing-based prior information to design an effective path detection threshold, thereby further refining CE accuracy.

To further validate the BER effectiveness of the proposed method, Figure 3 depicts the BER performance curves. For each given SNR, “LoS_based+ZF” achieves superior BER performance compared to “LS+ZF”, “LS_DFT+ZF”, and “Path_gains_based+ZF”. When SNR=12dB, the BER of “LoS_based+ZF” is $4.26\times 10^{-3}$, while those of “LS+ZF”, “LS_DFT+ZF”, and “Path_gains_based+ZF” all exceed $4.95\times 10^{-3}$. This result demonstrates that LoS sensing is effective in reducing the BER. The primary reason is that the utilization of LoS sensing-based prior information mitigates CE errors, thereby improving the BER performance. In Figure 3, the BER performance of both “LS_DD+ZF” and “Prop_PCC+ZF” is lower than that of “LS+ZF”, “LS_DFT+ZF”, “Path_gains_based+ZF”, “LoS_based+ZF”, and “OMP_based+ZF” for each given SNR. This indicates that “LS_DD+ZF” and “Prop_PCC+ZF” enhance the CE accuracy by leveraging echo sensing-based information and thus refine their BER performance. Furthermore, “Prop_PCC+ZF” achieves slightly superior BER performance compared to “LS_DD+ZF” for each specific SNR. For the case where SNR=12 dB, the BER of “Prop_PCC+ZF” is $2.42\times 10^{-4}$, whereas that of “LS_DD+ZF” is $2.55\times 10^{-4}$. This result demonstrates that the proposed method effectively integrates both LoS sensing and echo sensing information, thereby enhancing the CE performance. Consequently, “Prop_PCC+ZF” exhibits a discernible advantage in terms of BER performance.

**Figure 4:**
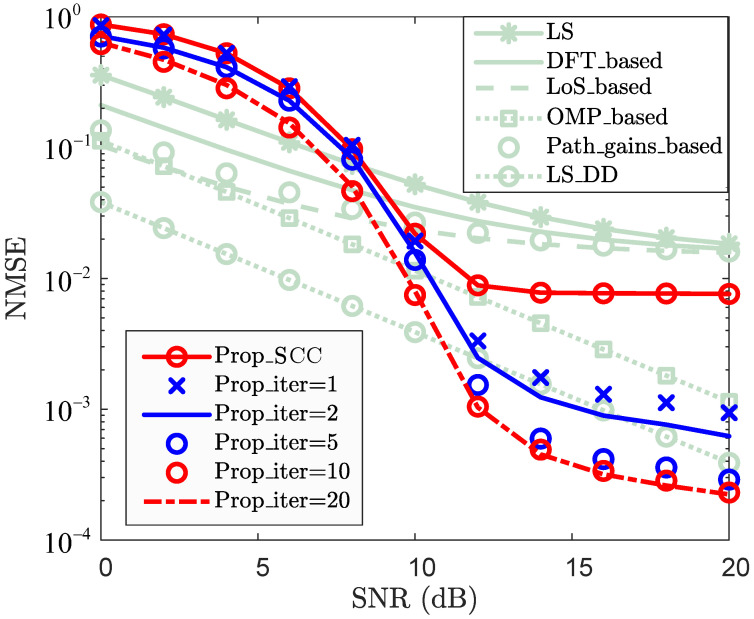
NMSE vs. SNR for SCC.

**Figure 5:**
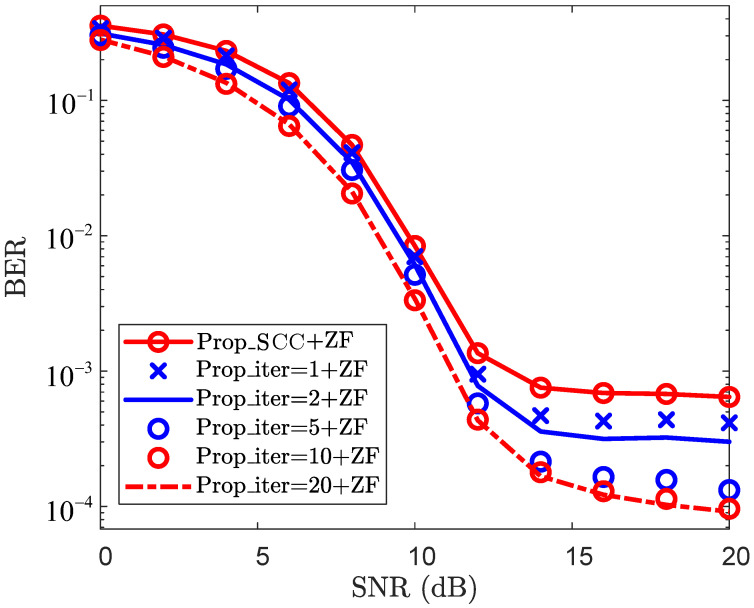
BER vs. SNR for SCC.

To evaluate the effectiveness of the SCC channel reconstruction, Figure 4 and Figure 5 present the NMSE and BER performance, respectively. To ensure a fair comparison in terms of NMSE performance, the same pilot-assisted CE scheme as used for the PCC is adopted as the baseline. Notably, the proposed method consumes only *M* resource elements as pilots. In contrast, the baseline methods utilize $MD/\left(\Delta_{M}\Delta_{D}\right)$ pilots for CE, with $\Delta_{M}$ and $\Delta_{D}$ denoting the pilot intervals along the subcarrier and OFDM symbol directions, respectively. This demonstrates that the pilot overhead of the baseline methods is approximately $D/\left(\Delta_{M}\Delta_{D}\right)$ times that of the proposed method (For the case where M=72, D=14, $\Delta_{M}=2$, and $\Delta_{D}=1$, the proposed scheme requires only 72 pilots, with a total number of resource elements amounting to 1008. In contrast, the baseline methods utilize 504 pilots, which is approximately seven times that of the proposed scheme. When the number of subcarriers and symbols increases, the pilot resources required by the proposed method remain limited to only one symbol (i.e., *M*). In contrast, the pilot resources are $MD/\left(\Delta_{M}\Delta_{D}\right)$. With an increased pilot interval, the pilot overhead of the proposed method is still smaller than that of the baseline methods). When SNR≥10dB, “Prop_SCC” achieves superior NMSE performance compared to the “LS+ZF”, “LS_DFT+ZF”, “Path_gains_based+ZF”, and “LoS_based+ZF”, this demonstrates that, in relatively high-SNR region, “Prop_SCC” not only reduces pilot overhead but also enhances channel reconstruction performance relative to the baseline methods. However, for the case where SNR≥10dB, “OMP_based” and “LS_DD” achieve smaller NMSEs than that of “Prop_SCC”. Nevertheless, “Prop_SCC” does not require an accurate sparsity as prior information compared to “OMP_based” and exhibits lower computational complexity even compared to “LS_DD”. The trade-off between channel reconstruction accuracy and iteration complexity in SCC is evaluated by comparing the accuracy for the cases where the iteration numbers are 1, 2, 10, 15, and 20. When SNR=12dB, the NMSEs of “Prop_SCC”, “Prop_iter=1”, “Prop_iter=2”, “Prop_iter=10”, “Prop_iter=15”, and “Prop_iter=20” are $8.86\times 10^{-3}$, $3.33\times 10^{-3}$, $2.47\times 10^{-3}$, $1.53\times 10^{-3}$, $1.05\times 10^{-3}$, and $1.03\times 10^{-3}$, respectively. This demonstrates that the NMSEs of the SCC channel reconstruction are improved as the number of iterations increases. When the number of iterations exceeds 10 and SNR≥10dB, “Prop_iter=15” and “Prop_iter=20” achieve better NMSE performance than “LS_DD”. This indicates that iterative processing significantly enhances the NMSE performance of the proposed scheme with increased computational complexity. However, increasing the number of iterations does not lead to a continual improvement in channel reconstruction accuracy. As shown in Figure 4, the accuracy improvement diminishes as iterations increase, with the NMSE performance being nearly identical at 15 and 20 iterations. This indicates that, although the reconstruction accuracy of the proposed method is significantly improved with a few initial iterations, it eventually converges as the number of iterations increases to 20. Consequently, a trade-off between reconstruction performance and computational complexity can be achieved. For instance, when one and two iterations are employed, “Prop_iter=1” and “Prop_iter=2” achieve similar performance to “LS_DD” in relatively high-SNR regions (e.g., SNR≥12dB) while maintaining similar computational complexity. Nonetheless, the pilot overhead of the proposed method remains substantially lower than that of the baseline schemes. Overall, the proposed approach achieves effective reconstruction accuracy with considerably reduced pilot overhead.

Figure 5 presents the BER performance of the SCC channel reconstruction. From Figure 5, the BER performance improves as the number of iterations increases, and this trend becomes more pronounced in relatively high-SNR regions (e.g., SNR≥12dB). For the case where SNR=8dB, the BERs of “Prop_SCC+ZF”, “Prop_iter=1+ZF”, “Prop_iter=2+ZF”, “Prop_iter=10+ZF”, “Prop_iter=15+ZF”, and “Prop_iter=20+ZF” are $4.69\times 10^{-2}$, $4.11\times 10^{-2}$, $3.49\times 10^{-2}$, $3.06\times 10^{-2}$, $2.12\times 10^{-2}$, and $2.05\times 10^{-2}$, respectively. In contrast, when SNR=16dB, the BERs of “Prop_SCC+ZF”, “Prop_iter=1+ZF”, “Prop_iter=2+ZF”, “Prop_iter=10+ZF”, “Prop_iter=15+ZF”, and “Prop_iter=20+ZF” are reduced to $6.89\times 10^{-4}$, $4.27\times 10^{-4}$, $3.15\times 10^{-4}$, $1.65\times 10^{-4}$, $1.29\times 10^{-4}$, and $1.22\times 10^{-4}$, respectively. Furthermore, the proposed method has 936 resource elements per time slot for symbol transmission, whereas the baseline methods only have 504. With the QPSK modulation, the proposed method transmits 1872 bits per time slot, with only 1008 bits being transmitted by the baseline methods. Under the same conditions, the proposed scheme achieves approximately 1.85 times the data transmission volume of the baseline methods. Thus, by leveraging sensing information and path-sharing to reconstruct the SCC channel, the proposed method reduces the pilot overhead of the SCC and thereby increases the data transmission rate.

### 4.4. Robustness Analysis

To verify the robustness of the proposed method, this section evaluates the impact of velocity *v*, the path count difference $\Delta_{L}$, and the carrier frequency of the SCC, respectively. Except for the parameters discussed in this section, all the other fundamental parameters remain consistent with those detailed in Section 4.1.

#### 4.4.1. Robustness Against Velocity *v*

**Figure 6:**
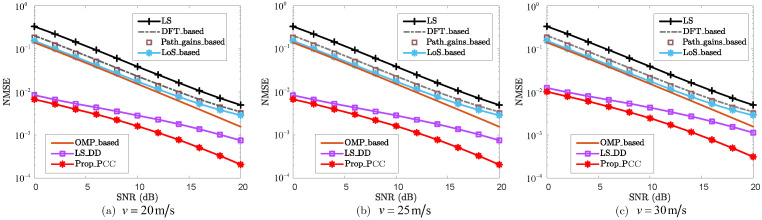
NMSE vs. SNR for PCC against velocity *v*, where v=20m/s, v=25m/s, and v=30m/s are considered.

**Figure 7:**
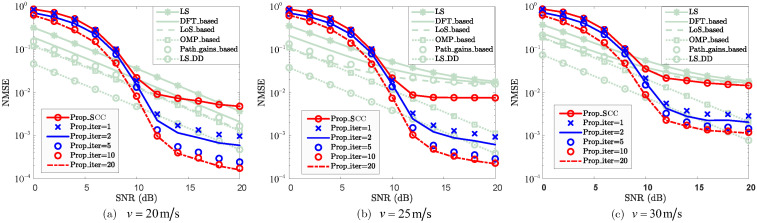
NMSE vs. SNR for SCC against velocity *v*, where v=20m/s, v=25m/s, and v=30m/s are considered.

In wireless communication systems, different velocities may affect the CE accuracy. To validate the robustness of the proposed method against velocity variations, Figure 6 and Figure 7 demonstrate the NMSE performance of the PCC and SCC against the impact of the variations in velocity, where v=20mmss, v=25mmss, and v=30mmss are considered.

From Figure 6, the proposed method, i.e., “Prop_PCC”, achieves a significantly lower NMSE than “LS”, “DFT_based”, “Path_gains_based”, “LoS_based”, “OMP_based”, and “LS_DD” for each given SNR and velocity. For the case where v=30mmss and SNR=16dB, the NMSEs of “LS”, “DFT_based”, “Path_gains_based”, “LoS_based”, “OMP_based”, and “LS_DD” are $1.07\times 10^{-2}$, $6.75\times 10^{-3}$, $6.19\times 10^{-3}$, $5.29\times 10^{-3}$, $3.93\times 10^{-3}$, and $2.06\times 10^{-3}$, respectively, while the NMSE of “Prop_PCC” is only $7.71\times 10^{-4}$. This demonstrates that the proposed method significantly enhances CE accuracy by effectively leveraging both LoS and echo sensing information. As the velocity increases from v=20mmss to v=30mmss, the Doppler shift also rises proportionally. As depicted in Figure 6, the NMSE performance remains consistent as the velocity increases. The primary reason is that, for any given velocity, the channel coherence time remains sufficiently longer than the time slot duration. According to the definition of channel coherence time, i.e., $T_{\mathrm{ct}}\approx 0.423/f_{D,\max}$, when the velocities are v=20mmss, v=25mmss, and v=30mmss, the coherence times of the PCC channel are approximately 3ms, 2.4ms, and 2ms, respectively. Consequently, the proposed method effectively enhances NMSE performance regardless of the variation in velocity.

In Figure 7, the NMSE performance of the SCC channel reconstruction with varying velocity is demonstrated. For the case where SNR=14dB, the NMSEs of “Prop_iter=15” for the velocities v=20mmss, v=25mmss, and v=30mmss are $3.88\times 10^{-4}$, $4.52\times 10^{-4}$, and $1.66\times 10^{-3}$, respectively. Correspondingly, the approximate channel coherence times of the SCC are 1.6ms, 1.3ms, and 1.1ms for the velocities of v=20mmss, v=25mmss, and v=30mmss, respectively. The NMSE performance of the SCC channel reconstruction deteriorates as the velocity increases due to the increased Doppler shift interference. Nevertheless, the reconstruction performance of the SCC achieves NMSE performance comparable to that of the baseline methods for each given velocity and SNR, especially in relatively high-SNR regions (e.g., SNR≥10dB). Especially, the proposed method requires significantly lower pilot overhead compared to the baseline methods, making it highly attractive for reconstructing the SCC channels.

#### 4.4.2. Robustness Against Path-Number Difference $\Delta_{L}$

**Figure 8:**
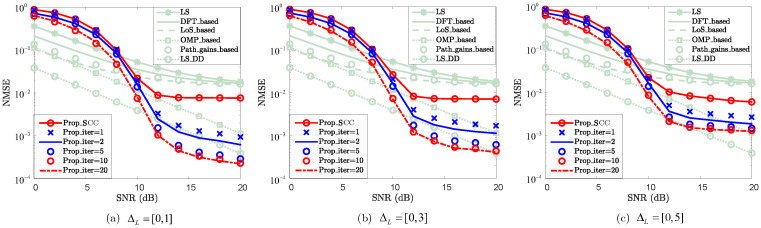
NMSE vs. SNR for SCC against path-number difference $\Delta_{L}$, where $\Delta_{L}=\left[0,1\right]$, $\Delta_{L}=\left[0,3\right]$, and $\Delta_{L}=\left[0,5\right]$ are considered.

To evaluate the impact of $\Delta_{L}$, the NMSE performance of the SCC reconstruction is validated, where the cases of $\Delta_{L}=\left[0,1\right]$, $\Delta_{L}=\left[0,3\right]$, and $\Delta_{L}=\left[0,5\right]$ are considered. As shown in Figure 8, the NMSE performance of the SCC reconstruction degrades as $\Delta_{L}$ increases. The decreased $\Delta_{L}$ and the presence of LoS sensing jointly mitigate this performance loss. For the case where SNR=12dB, the NMSEs of “Prop_SCC” for $\Delta_{L}=\left[0,1\right]$, $\Delta_{L}=\left[0,3\right]$, and $\Delta_{L}=\left[0,5\right]$ are $8.26\times 10^{-3}$, $8.86\times 10^{-3}$, and $1.03\times 10^{-2}$, respectively. This is primarily due to the introduction of additional unreconstructed path errors in the SCC channel during the path-sharing reconstruction stage. It is worth noting that similar NMSE performance is achieved for different values of $\Delta_{L}$. The main reason is that the existence of LoS paths occupies the majority of the channel energy, enabling the “Prop_SCC” to maintain similar performance and ensuring the effectiveness of the reconstruction. Although such errors are partially mitigated by increasing the number of iterations, they still degrade the reconstruction performance. Increasing $\Delta_{L}$ accelerates convergence but results in fewer errors being eliminated. This is because the iterative processing introduces more non-path reconstruction errors, which degrade the final performance as $\Delta_{L}$ increases. Nonetheless, the proposed scheme achieves channel reconstruction performance comparable to the baseline method with lower pilot overhead, making it practical.

#### 4.4.3. Robustness Against Carrier Frequencies of SCCs

**Figure 9:**
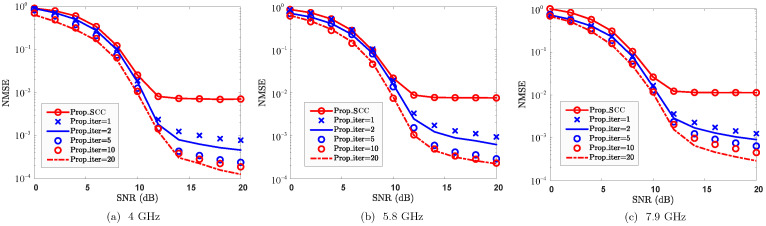
NMSE vs. SNR against carrier frequencies of SCCs, where 4 GHz, 5.8 GHz, and 7.9 GHz are considered.

CA systems typically operate with multiple SCC frequency bands, and therefore the proposed method also supports this feature. To validate the effectiveness of SCC reconstruction against the impact of carrier frequency, Figure 9 plots the NMSE performance, where 4 GHz, 5.8 GHz, and 7.9 GHz are considered for the SCC bands. From Figure 9, the proposed method effectively reconstructs the SCC channel across all the evaluated frequency bands. Furthermore, the reconstruction performance improves as the number of iterations increases. However, the reconstruction accuracy slightly degrades as the carrier frequency of the SCC increases. The primary reason for this is that a higher carrier frequency results in an increase in Doppler shift for the paths, which amplifies the reconstruction error introduced by non-shared paths and consequently degrades the overall reconstruction performance. Furthermore, as the Doppler shift increases, the channel correlation is weakened correspondingly. When the carrier frequencies are 4 GHz, 5.8 GHz, and 7.9 GHz, the channel coherence times of the SCC are approximately 1.3ms, 0.875ms, and 0.642ms, respectively. As the first symbol is utilized for path gain reconstruction, the increase in carrier frequency thereby increases its reconstruction errors due to larger Doppler shifts and reduced coherence time. Despite the performance degradation caused by increasing carrier frequency, the proposed method still effectively reconstructs the SCC channel with low pilot overhead. From a resource efficiency perspective, the proposed approach presents a highly attractive solution for channel reconstruction in CA systems.

## 5. Conclusions

In this paper, we investigate CE in UAV-assisted OFDM systems by leveraging LoS and echo sensing with CA. By exploiting the prominent LoS characteristics and echo sensing information in UAV communication scenarios, sensing-assisted prior information is established. Based on this prior information, the CE accuracy of the PCC in CA is significantly improved. Inspired by the LoS path, a method for reconstructing the SCC channel is proposed by exploiting the path-sharing property between the PCC and SCCs. Furthermore, this path sharing is extended to NLoS paths for enhancing the reconstruction of the SCC channel. By leveraging the path-sharing property between the PCC and SCCs, the pilot overhead of the SCCs is significantly reduced. To reconstruct non-shared paths and mitigate reconstruction errors, an iterative processing scheme is developed. This scheme enhances the accuracy of the SCC channel reconstruction. The simulation results demonstrate the effectiveness of the proposed method in enhancing CE accuracy for both PCCs and SCCs. Against parameter variations, the proposed method also exhibits its robustness. In our future work, we will investigate the performance evaluation, benchmark testing, and verification of measured data in real UAV-assisted communication scenarios.

## Figures and Tables

**Table 1:** Summary of the related works.

Literature	Pros	Cons
[5]	Tailored tensor models across different UAV-assisted communication scenarios	Overlook the dominant LoS characteristics inherent in UAV scenarios
[6]	Deep learning-based enhanced bandwidth efficiency in time-varying environments	
[7]	Multi-resolution deep neural network-based computational efficiency	
[14]	Gridless compressed sensing (CS)-based beam squint mitigation	
[15]	Hybrid parametric/non-parametric CE scheme incorporating UAV state-space information	
[16]	LoS sensing-based denoising threshold for LoS/non-line-of-sight (NLoS) detection	Ignore the wireless sensing-based enhancement schemes
[21,22,23,24,25,26,27,28,29,30,31]	Sensing-based CE enhancement schemes	The advantages in the UAV scenarios have not been fully explored
	Provide valuable insights for CE in UAV-assisted systems	
[32]	Channel correlation across different component carriers (CCs)	Overlook the LoS characteristics and wireless sensing-assisted schemes
[33]	Subchannel selection strategy for CA-OFDM systems	
[34]	Adaptive pilot interval and power allocation for CE	
[35]	CA-OFDM experimental testbed	
[36]	Enhanced ISAC signal design and sensing performance	
Proposed method	LoS and echo sensing-based prior information for communication-centric ISAC design	
	Sensing-aided CE enhancement scheme in UAV-assisted communication scenarios	
	Path sharing-based channel reconstruction scheme between the PCC and SCCs	

**Table 2:** Abbreviations and descriptions.

Abbreviation	Description	Abbreviation	Description
5G	5th-Generation	6G	6th-Generation
AoA	Angle-of-Arrival	AoD	Angle-of-Departure
AWGN	Additive White Gaussian Noise	BER	Bit-Error Rate
CA	Carrier Aggregation	CA-CFAR	Cell-Averaging Constant False Alarm Rate
CC	Component Carrier	CE	Channel Estimation
CFR	Channel Frequency Response	CIR	Channel Impulse Response
CM	Complex Multiplication	CP	Cyclic Prefix
CS	Compressed Sensing	CSI	Channel State Information
dB	Decibels	DD	Delay-Doppler
DFT	Discrete Fourier Transform	FFT	Fast Fourier Transform
gBS	ground Base Stations	IDFT	Inverse Discrete Fourier Transform
IRS	Intelligent Reflecting Surface	ISAC	Integrated Sensing and Communication
ISFFT	Inverse Symplectic Finite Fourier Transform	ISI	Inter-Symbol Interference
LoS	Line-of-Sight	LS	Least Squares
MIMO	Multiple-Input Multiple-Output	MMSE	Minimum Mean Square Error
MUSIC	Multiple Signal Classification	NLoS	Non-Line-of-Sight
NMSE	Normalized Mean Square Error	OFDM	Orthogonal Frequency Division Multiplexing
PCC	Primary Component Carrier	PDP	Power Delay Profile
QPSK	Quadrature Phase Shift Keying	SCC	Secondary Component Carrier
SD	Symbol Detection	SFFT	Symplectic Finite Fourier Transform
SNR	Signal-to-Noise Ratio	TDL	Tapped Delay Line
UAV	Unmanned Aerial Vehicle	U2G	UAV-to-Ground

**Table 3:** Summary of the key results.

Observed Anomaly/Alignment	Implications	Potential Application Scenarios
“Prop_PCC” achieves the optimal NMSE performance compared to the baseline method.	“Prop_PCC” incorporates LoS sensing and echo sensing-based prior information to design an effective path detection threshold, thereby further refining CE accuracy.	High-precision control links for autonomous UAV swarms in urban environments.
“Prop_PCC+ZF” exhibits a discernible advantage in terms of BER performance compared to the baseline method.	“Prop_PCC+ZF” enhances the CE accuracy by leveraging LoS sensing and echo sensing-based information, thereby refining BER performance.	High-speed aerial video transmission requiring low bit-error rates.
“Prop_SCC” achieves superior NMSE performance compared to “LS+ZF”, “LS_DFT+ZF”, “Path_gains_based+ZF”, and “LoS_based+ZF” in relatively high-SNR region.	“Prop_SCC” not only reduces pilot overhead but also enhances channel reconstruction performance relative to the baseline methods.	Wideband CA systems for real-time HD mapping and sensor data fusion.
Increasing the number of iterations does not lead to a continual improvement in channel reconstruction accuracy.	The reconstruction accuracy of the proposed method is significantly improved with a few initial iterations; it eventually converges as the number of iterations increases. A trade-off between reconstruction performance and complexity can be achieved.	Enables the design of low-complexity energy-efficient transceivers for computation-limited UAV platforms.
Robustness Against Velocity *v*	“Prop_PCC” effectively enhances NMSE performance regardless of the variation in velocity. “Prop_SCC” requires significantly lower pilot overhead compared to the baseline methods, making it highly attractive for reconstructing the SCC channels.	High-mobility applications such as fast-moving UAVs for emergency response or delivery.
Robustness Against Path Count Difference $\Delta_{L}$	The existence of LoS paths occupies the majority of the channel energy, enabling the “Prop_SCC” to maintain similar performance and ensuring the effectiveness of the reconstruction.	Non-uniform scattering environments (e.g., open fields vs. dense urban areas).
Robustness Against Carrier Frequency of SCC	The increase in carrier frequency thereby increases its reconstruction errors due to larger Doppler shifts and reduced coherence time.	Guides the practical deployment in multi-band CA systems, suggesting prioritization of lower frequencies for critical control channels.

## Data Availability

The data presented in this study are available on request from the corresponding author.

## Appendix A. Sensing-Based Prior Information Derivation

With the prior information set I, the prior information of multipath delay and Doppler spreads is developed for CE. According to I, we can search the first arrival path delay and the maximum path delay, i.e.,

(A1) $$\left\{\begin{array}{c} \tau_{\min}=\underset{{\hat{\tau }}_{\bar{p}}}{\arg\min}\left\{{\hat{\tau }}_{\bar{p}}\right\}\\ \tau_{\max}=\underset{{\hat{\tau }}_{\bar{p}}}{\arg\max}\left\{{\hat{\tau }}_{\bar{p}}\right\} \end{array}\right.,$$

where $\tau_{\min}$ and $\tau_{\max}$ denote the first arrival path delay and the maximum path delay, respectively. Then, the delay spread $\sigma_{\tau }$ is calculated by

(A2) $$\sigma_{\tau }=\tau_{\max}-\tau_{\min}.$$

Correspondingly, the maximum Doppler frequency shift is expressed as

(A3) $$f_{D,\max}=\underset{{\hat{f}}_{D,\bar{p}}}{\arg\max}\left\{\left|{\hat{f}}_{D,\bar{p}}\right|\right\}.$$

Thus, the estimated Doppler spread $\sigma_{\nu }$ satisfies

(A4) $$\sigma_{\nu }=f_{D,\max}-\left(-f_{D,\max}\right)=2f_{D,\max}.$$

According to Equations (A2) and (A4), the ranges of delay and Doppler shift of the $\bar{p}$-th path are bounded by

(A5) $$\left\{\begin{array}{c} 0\leq {\hat{\tau }}_{\bar{p}}\leq \tau_{\max}-\tau_{\min}\\ -f_{D,\max}\leq {\hat{f}}_{D,\bar{p}}\leq f_{D,\max} \end{array}\right..$$

By denoting $T_{\tau }$ and $T_{\nu }$ as the delay and Doppler resolutions, respectively, we have

(A6) $$\left\{\begin{array}{c} T_{\tau }=\frac{1}{K\Delta f_{\mathrm{uc}}}\\ T_{\nu }=\frac{\Delta f_{\mathrm{uc}}}{S} \end{array}\right..$$

Subsequently, the index ranges of delay and Doppler grids for the $\bar{p}$-th path are given by

(A7) $$\left\{\begin{array}{c} 0\leq \left\lceil \frac{{\hat{\tau }}_{\bar{p}}}{T_{\tau }}\right\rceil \leq \left\lceil \frac{\sigma_{\tau }}{T_{\tau }}\right\rceil \\ -\left\lceil \frac{f_{D,\max}}{T_{\nu }}\right\rceil \leq \left\lceil \frac{{\hat{f}}_{D,\bar{p}}}{T_{\nu }}\right\rceil \leq \left\lceil \frac{f_{D,\max}}{T_{\nu }}\right\rceil \end{array}\right..$$
